# Differential Risk Factors for Lacunar Stroke Depending on the MRI (White and Red) Subtypes of Microangiopathy

**DOI:** 10.1371/journal.pone.0044865

**Published:** 2012-09-14

**Authors:** Jae-Hyun Park, Sookyung Ryoo, Suk Jae Kim, Gyeong-Moon Kim, Chin-Sang Chung, Kwang Ho Lee, Oh Young Bang

**Affiliations:** Department of Neurology, Samsung Medical Center, Sungkyunkwan University School of Medicine, Seoul, Korea; University of Cambridge, United Kingdom

## Abstract

**Background:**

Leukoaraiosis and cerebral microbleeds (CMB), which represent cerebral microangiopathy, commonly coexist in patients with acute lacunar stroke. Since they may have different impacts on stroke prognosis and treatment, it is important to know the factors associated with leukoaraiosis-predominant *vs.* CMB-predominant microangiopathies.

**Methods:**

We prospectively recruited 226 patients with acute lacunar infarction and divided them into four groups according to the Fazekas’ score and the presence of CMB: mild, red (predominant CMB), white (predominant leukoaraiosis) and severe microangiopathy groups. For comparison, we also evaluated 50 patients with intracerebral hemorrhage (ICH). We evaluated the clinical and laboratory findings of microangiopathy subtypes in patients with acute lacunar stroke and then compared them with those of primary ICH.

**Results:**

The risk factor profile was different among the groups. Patients with acute lacunar infarct but mild microangiopathy were younger, predominantly male, less hypertensive, and more frequently had smoking and heavy alcohol habits than other groups. The risk factor profile of red microangiopathy was similar to that of ICH but differed from that of white microangiopathy. The subjects in the white microangiopathy group were older and more frequently had diabetes than those in the red microangiopathy or ICH group. After adjustments for other factors, age [odds ratio (OR) 1.13; 95% confidence interval (CI) 1.08–1.18; *p*<0.001] and diabetes (OR 2.28; 95% CI 1.02–5.13; *p* = 0.045) were independently associated with white microangiopathy, and age (OR 1.05; 95% CI 1.01–1.08; *p* = 0.010) was independent predictor for red microangiopathy compared to mild microangiopathy.

**Conclusion:**

Patients with acute lacunar infarction have a different risk factor profile depending on microangiopathic findings. Our results indicate that diabetes may be an one of determinants of white (leukoaraiosis-predominant) microangiopathy, whereas smoking and alcohol habits in relatively young people may be a determinants of mild microangiopahic changes in patients with lacunar infarction.

## Introduction

Little attention has been paid to concomitant silent white matter ischemic or bleeding changes in acute lacunar stroke patients. However, both leukoaraiosis and cerebral microbleeds (CMB) are very frequently observed in patients with lacunar stroke subtypes.

Leukoaraiosis and CMB may have different impacts on stroke prognosis and treatment. Leukoaraiosis describes diffuse white matter abnormalities on neuroimaging and is associated with vascular risk factors, recurrent stroke, and cognitive impairment. A multicenter, follow-up magnetic resonance imaging (MRI) (Leukoaraiosis and Disability) study showed that baseline leukoaraiosis and lacunes predicted both progression of leukoaraiosis and new lacunes [1]. Antiplatelet agents have been used to prevent lacunar stroke or progression of leukoaraiosis. A recent randomized controlled trial showed the usefulness of cilostazol in preventing recurrence of vascular events, particularly in patients with lacunar stroke [2]. In contrast, hemorrhagic stroke more frequently occurred after lacunar stroke than after non-lacunar stroke [3]. CMB are associated with intracerebral hemorrhage (ICH) in patients with lacunar stroke or ICH that occurs while taking antithrombotic agents [4].

Although their significance for treatment decisions and patient management remains to be defined, it is important to know the factors associated with leukoaraiosis-predominant (“white”) *vs.* CMB-predominant (“red”) microangiopathies [5]. to minimize both progression of ischemic damages and risk of bleeding complications. We hypothesized that risk factor profiles may be different between red *vs*. white microangiopathy. In this study, we evaluated the clinical and laboratory findings in patients of various subtypes of microangiopathy. In addition, we also compared vascular risk factors and white matter changes between patients with microangiopathy and those with primary ICH.

## Materials and Methods

### Patient

Our analysis was performed on data collected from a prospective registry of patients who were admitted for acute cerebral infarction to a university medical center from January 2008 through October 2011 and patients who were admitted for acute primary ICH from September 2009 through April 2011. The inclusion criteria of acute ischemic stroke for this study were: (a) focal neurologic deficits that presented within 7 days of the onset of symptoms and (b) acute ischemic lesions on diffusion-weighted image (DWI). Among 1740 patients who met inclusion criteria of ischemic stroke, 230 (13.2%) patients were classified as having evident small-artery occlusion (SAO) stroke etiology by the Stop Stroke Study Trial of Org 10172 in Acute Stroke Treatment classification (SSS-TOAST) [6]. The stroke mechanisms were diagnosed by consensus of two neurologists (J.H.P and S.R.). Among 230 patients classified as SAO, 4 patients with strictly lobar CMB were excluded because they might have cerebral amyloid angiopathy. Therefore 226 patients with acute lacunar infarction were finally included. The inclusion criteria of the acute ICH group were: (a) focal neurologic deficits or headache that presented within 7 days before admission and (b) acute ICH on brain computed tomography (CT) and MRI. Among 111 patients with acute ICH, 61 patients who had other causes were excluded and 50 patients with primary ICH were finally included. The local Institutional Review Board approved this study, and all patients gave their consent to participate in this study.

#### Workups

We collected clinical information, including age, sex, and vascular risk factors, and all patients underwent diagnostic testing that included routine blood tests, electrocardiography, cardiac telemetry for at least 24 hours, and echocardiography. Routine blood tests included complete blood cell count, erythrocyte sedimentation rate (ESR), chemistry profile [blood glucose level, hemoglobin A1c, liver function test, creatinine, glomerular filtration rate (calculated from creatinine level), blood urea nitrogen, high sensitivity C-reactive protein], lipid profile [total cholesterol, triglyceride (TG), low-density lipoprotein (LDL) cholesterol, high-density lipoprotein cholesterol, lipoprotein (a)] and coagulation profile (prothrombin time, activated partial thromboplastin time, D-dimer, fibrinogen). We also collected urine lab including albumin, creatinine and albumin-to-creatinine ratio. Hemostatic markers of prothrombotic tendency, including antiphospholipid antibodies, were measured in patients who were younger than 50 years.

Our definitions of vascular risk factors were as follows. (1) Hypertension was deemed present when the patient had been undergoing treatment with antihypertensive agents, or their blood pressure was either ≥140 mmHg systolic or ≥90 mmHg diastolic on at least two occasions after the acute phase of their ischemic stroke. (2) Diabetes mellitus was deemed present when the patient had been receiving medication for diabetes, had an elevated fasting glucose level ≥126 mg/dL (7.0 mmol/L) or a 2-hour plasma glucose ≥200 mg/dL (11.1 mmol/L) during their oral glucose tolerance test, or had a plasma glucose level ≥200 mg/dL (11.1 mmol/L), along with classic symptoms of hyperglycemia, a hypoglycemic crisis, or a hemoglobin A1c >6.5% [7]. (3) Dyslipidemia was determined to be present if the patient had been taking lipid-lowering agents or had total cholesterol >240 mg/dL (6.21 mmol/L), TG>200 mg/dL (2.26 mmol/L), or LDL cholesterol >160 mg/dL (4.14 mmol/L). (4) Current smokers were those who regularly smoked at least one cigarette per day when admitted to the center. (5) Heavy alcohol consumption was defined as consumption of more than 180 g/week, and social drinking was defined as consumption of less than 180 g/week.

### Imaging Analysis

All participants underwent MRI on a 3-T system (Achieva, Philips Medical System, Best, the Netherlands) including DWI (repetition time 2500 ms, echo time 75 ms, matrix number 128×128, 2 b values of 0 and 1000 s/mm^2^, slice thickness 5 mm, interslice gap 2 mm, 20 axial slices, and field of view 240 mm), T2 fluid-attenuated inversion recovery (FLAIR; using a fast-spin echo sequence with repetition time/echo time = 11000/125 ms, inversion time = 2800 ms, and a 320×252 matrix), gradient-echo (GRE; 645 ms repetition time, 16 ms echo time, 18° flip angle, 256×256 matrix, slice thickness 5 mm, interslice gap 2 mm, 20 axial slices, and field of view 240 mm), and T1- and T2-weighted images. We also obtained three-dimensional, time-of-flight magnetic resonance angiography (MRA) of the intracranial arteries (repetition time 25 ms, echo time 3.5 ms, 80 slices of 0.45 mm thickness over contiguous sampling, 20° flip angle, a 880×450 matrix, and field of view 170 mm) and gadolinium-enhanced MRA of the extracranial arteries from all patients.

We measured neuroimaging indicators of microangiopathies (CMB and leukoaraiosis) in all patients. Leukoaraiosis was defined as a hyperintense white matter lesion on T2-FLAIR images lacking prominent hypointensity on T1-weighted images. Leukoaraiosis was graded using a modification of Fazekas’ method [8]. We considered CMB to be small (≤10 mm), homogeneous, round foci of low signal intensity that were distinct from other potential mimics such as iron or calcium deposits, bone, or vessel flow voids on GRE sequences and manually counted the number of CMB for each patient [9]. Two experienced neurologists (JHP, SJK), who were blinded to other patient data, measured the grade of leukoaraiosis and number of CMB. We also counted the number of steno-occlusive lesions in non-relevant intracranial arteries, regarded as indicators of the intracranial atherosclerotic stenosis (ICAS) burden.

**Figure 1:**
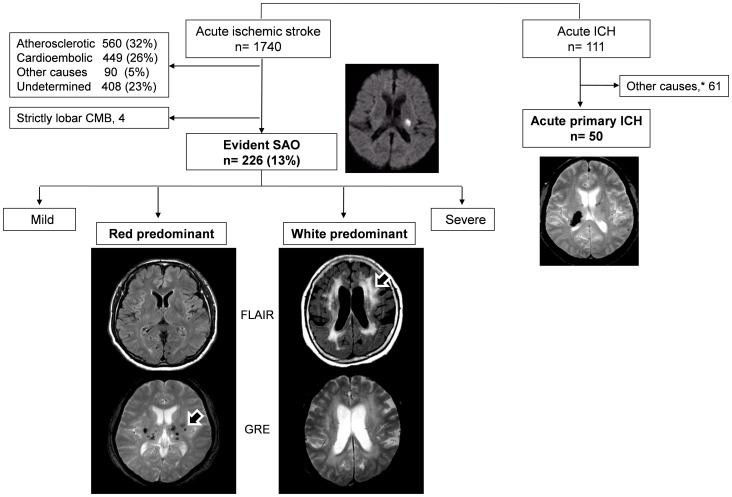
Patient selection . *The other causes indicate brain tumor, vascular malformation, Moyamoya disease, coagulopathy such as disseminated intravascular coagulation, antithrombotic medication, trauma, and hemorrhagic transformation of ischemic stroke.

We divided patients into four groups according to their grade of leukoaraiosis (Fazekas’ score) and number of CMB. Patients were grouped as follows: (1) ‘mild’ microangiopathy group (n = 112) – patients with Fazekas’ score ≤2 and CMB = 0, (2) ‘red’ group of patients who had predominant CMB (n = 45) – Fazekas’ score ≤4 and CMB ≥1, (3) ‘white’ group of patients who had predominant leukoaraiosis (n = 48) – Fazekas’ score ≥3 and CMB = 0, and (4) ‘severe’ microangiopathy group (n = 25) – Fazekas’ score ≥5 and CMB ≥1. Figure 1 shows patients’ selection and examples of the red and white groups.

### Statistical Analysis

We analyzed the differences between the groups of microangiopathy using Pearson’s χ^2^ test or Fisher’s exact test for categorical variable. For continuous variables, one-way analysis of variance (ANOVA) or Kruskal-Wallis test was used. Multiple group comparisons were performed using Tukey’s post-hoc test (ranked data were used for nonparametric variables). Multivariate logistic regression analysis was performed to predict the independent contribution of factors for red or white microangiopathy compared to mild microangiopathy. Variables significant at *p*<0.2 on univariate analyses were considered explanatory variables and were entered together into the multivariate model. Data were processed using the PASW Statistics 18 (SPSS Inc, Chicago, IL, USA) software package. A *p*-value <0.05 was considered statistically significant.

## Results

### General Characteristics

A total of 226 lacunar stroke and 50 primary ICH patients were included in this study: 139 men and 87 women, mean age 63.9±12.1 years (range, 33–95) in the lacunar stroke group; 30 men and 20 women, mean age 60.7±13.4 years (range, 31–90) in the ICH group.

**Figure 2:**
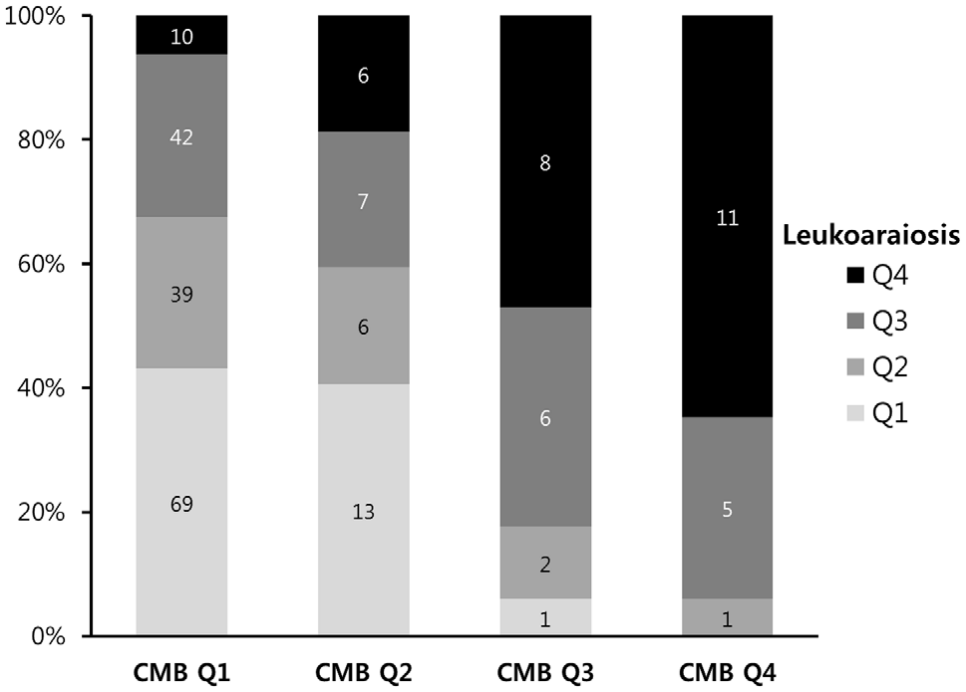
Correlation between the degree of leukoaraiosis and cerebral microbleeds. Leukoaraiosis quartiles were categorized as follows: Q1, Fazekas’ score = 0 to 1; Q2, 2; Q3, 3 to 4; and Q4, 5 to 6. Cerebral microbleeds (CMB) quartiles were categorized as follows: Q1, number of CMB = 0; Q2, 1 to 2; Q3, 3 to 10; and Q4, over 11.

For the imaging markers of microangiopathy, the rate of agreement between two neurologists was 87.3% for leukoaraiosis and 91.1% for CMB, and a consensus was reached in all cases of discrepancy. The severity of leukoaraiosis and number of CMB were significantly correlated with each other (rho = 0.402, *p*<0.001) (Figure 2). Fazekas’ score of the red microangiopathy group was similar to the ICH group (Table 1). CMB were frequently observed in the both ICH and red microangiopathy group, similarly. There was no difference in terms of acute infarct size on DWI or the presence of asymptomatic intracranial atherosclerotic stenosis among subtypes.

**Table 1:** Comparison of risk factors in acute lacunar infarction according to subtypes of microangiopathy and ICH.

	Subtypes of microangiopathy in acute lacunar stroke				*p*-value		ICH (n = 50)	*p*-value	
	Mild (n = 112)	Red(n = 41)	White(n = 48)	Severe (n = 25)	Overall	Red *vs.* white		Red *vs.* ICH	White *vs.* ICH
MRI findings									
Fazekas’ score, median (IQR)	1 (0–2)	2 (1–3)	3 (3–4)	6 (5–6)	<0.001	<0.001	2 (0–3)	0.524	<0.001
Microbleeds, median (IQR)	–	2 (1–3)	–	9 (3–21)	<0.001	<0.001	1 (0–3)	0.008	<0.001
Lobar microbleeds	–	0 (0–1)	–	3 (1–6)	–	–	0 (0–1)	0.950	–
Deep microbleeds	–	2 (1–3)	–	4 (2–15)	–	–	1 (0–3)	0.004	–
DWI size (diameter, mm)	11.1±4.1	10.8±3.4	11.3±3.3	11.2±2.9	0.948	0.523			
Asymptomatic ICAS, n (%)	21 (18.8)	5 (12.2)	11 (22.9)	5 (20)	0. 629	0.189			
Risk factor profile, n (%)									
Age, y (mean±SD)	58.8±10.5	63.9±12.1	70.2±9.1	74.4±11.3	<0.001	0.006	60.7±13.4	0.246	<0.001
Female	39 (34.8)	11 (26.8)	19 (39.6)	18 (72)	0.002	0.205	20 (40)	0.187	1.000
Hypertension	60 (53.6)	28 (68.3)	33 (68.8)	20 (80)	0.038	0.963	41 (82)	0.129	0.255
Diabetes mellitus	37 (33)	9 (22.0)	22 (45.8)	4 (16)	0.028	0.018	10 (20)	0.820	0.023
Hyperlipidemia	30 (26.8)	7 (17.1)	7 (14.6)	4 (16)	0.244	0.748	5 (10)	0.176	0.513
Prior ischemic stroke/TIA	9 (8.0)	4 (9.8)	9 (18.8)	4 (16)	0.220	0.231	5 (10)	1.000	0.432
Prior history of ICH	–	2 (4.9)	–	2 (8.0)	0.013	0.122	3 (6.0)	1.000	0.170
Current smoker	45 (40.2)	12 (29.3)	8 (16.7)	1 (4)	<0.001	0.156	11 (22.4)	0.460	0.946
Heavy alcohol consumption	28 (25)	10 (24.4)	8 (16.7)	1 (4)	0.098	0.366	13 (26)	1.000	0.521

TIA indicates transient ischemic attack; ICH, intracerebral hemorrhage; IQR, interquartile range; ICAS, intracranial atherosclerotic stenosis.

### Risk Factor Profiles

First, we compared risk factors among subtypes of microangiopathy and ICH groups (Table 1). Age was different among groups; specifically, the patients in the mild microangiopathy group were younger than those in other groups (*p<*0.01 in all the cases). Female sex (*p* = 0.002) and hypertension (*p* = 0.038) were significantly most frequent in the severe microangiopathy group. The incidence of diabetes was significantly higher in the white microangiopathy group (*p* = 0.028). Patients with prior history of ICH were only observed in the red and severe microangiopathy groups. The highest number of current smoker was in the mild microangiopathy group, and the lowest number was in the severe group (*p*<0.001). There was a tendency (*p* = 0.098) that heavy alcohol consumption was most frequent in the mild microangiopathy group and least frequent in the severe group. Hyperlipidemia and prior history of ischemic stroke were not different among groups.

The comparison between red and white microangiopathy revealed that the white group was older (mean ± SD: 70.2±9.1 *vs*. 63.9±12.1, *p* = 0.006) and more often had diabetes (45.8% *vs*. 22.0%, *p* = 0.018) than the red microangiopathy group. We also compared the risk factors between the ICH and red or white microangiopathy groups. There was no difference between the ICH and red microangiopathy group. On the contrary, the patients with ICH were younger (60.7±13.4 *vs*. 70.2±9.1, *p*<0.001) and fewer had diabetes (20% *vs*. 45.8%, *p* = 0.023) than in the white microangiopathy group.

### Laboratory Findings

Glucose at admission, hemoglobin A1c, lipoprotein (a), ESR, fibrinogen, and D-dimer levels were different among groups (Table 2). Glucose at admission and hemoglobin A1c levels were higher in the white microangiopathy group than in other groups (*p<*0.01 in both cases). We then compared laboratory findings between the red and white microangiopathy groups by t-test with Bonferroni correction. The white microangiopathy group had higher glucose at admission (median: 140 *vs*. 122, *p* = 0.036), hemoglobin A1c (7.0±1.73 *vs*. 6.18±1.08, *p* = 0.019), and d-dimer levels (median: 0.43 *vs*. 0.36, *p* = 0.032).

**Table 2:** Comparison of laboratory findings in ischemic stroke according to subtypes of microangiopathy.

	Subtypes of microangiopathy in acute lacunar stroke				*p*-value	
	Mild (n = 112)	Red (n = 41)	White (n = 48)	Severe (n = 25)	Overall	Red *vs*. White
Glucose at admission, mg/dL	123 (109–163)	122 (107–146)	140 (108–223)	112 (97–136)	0.009	0.036
Hemoglobin A1c, %	6.49±1.49	6.18±1.08	7.00±1.73	5.79±1.07	0.008	0.019
Cholesterol, mg/dL	190.4±42.7	185.6±43.7	184.4±37.3	179.6±50.6	0.646	0.883
Triglyceride, mg/dL	135 (86–215.5)	135 (81.5–176)	132 (95–204)	102 (85–164.5)	0.527	0.648
HDL-cholesterol, mg/dL	46.7±21.2	46.15±12.7	45±12	43.7±10.2	0.848	0.652
LDL-cholesterol, mg/dL	121.7±32.2	113.5±32.8	112.7±28.8	113±39.2	0.262	0.894
Lipoprotein (a), mg/dL	19.3 (8.7–33.9)	24.3 (12.3–49.7)	23.9 (13.9–45.2)	40.6 (21.3–52.4)	0.025	0.813
ESR, mm/hr	13 (6–21.5)	21 (9.5–33)	20 (7.5–34.75)	22 (11.5–33.5)	0.003	0.908
Fibrinogen, mg/dL	299 (263–345)	337 (274–421)	340 (283–413)	360 (306–404)	0.002	0.897
D-dimer, µg/mL	0.26 (0.22–0.39)	0.36 (0.24–0.58)	0.43 (0.36–0.74)	0.52 (0.32–1.34)	<0.001	0.032
hsCRP, mg/dL	0.08 (0.04–0.18)	0.11 (0.04–0.31)	0.11 (0.06–0.58)	0.24 (0.05–0.47)	0.073	0.334
Urine albumin-to-creatinine ratio	9.2 (4.3–41.8)	10.4 (5.2–45.9)	16.1 (6.2–130.2)	17.9 (7.1–95.2)	0.169	0.3 73

Values are mean ± SD or median (Interquartile range).

HDL indicates high-density lipoprotein; LDL, low-density lipoprotein; hsCRP, high-sensitivity CRP; DWI, diffusion-weighted MRI.

Lipoprotein (a) levels were higher in the severe group compared to the mild microangiopathy group (*p* = 0.025). ESR and fibrinogen levels were lower in the mild microangiopathy group compared to other groups (*p<*0.05 in all the cases). Because age is associated with ESR and fibrinogen levels [10], we additionally compared ESR and fibrinogen levels among groups using Analysis of Covariance (ANCOVA) tests after adjusting to age. They still lower in mild microangiopathy group than severe group (ESR: *p*-value = 0.041, fibrinogen: *p*-value = 0.001). Serum D-dimer levels were higher in the white and severe microangiopathy groups compared to the mild microangiopathy group (*p*<0.05 in all cases). Correlation analysis showed that age and laboratory finding of elevated fibrinogen, ESR, D-dimer, and lipoprotein (a) levels were correlated with the severity of microangiopathies (both Fazekas’ score and the number of CMB) (Table S1). Moreover, there was a relationship between risk factors (age and hypertension) and laboratory findings (Table S2 and S3).

The levels of cholesterol and urine albumin-creatinine ratio were not different among the groups.

### Multivariate Testing

Multivariate logistic regression analysis was performed to further evaluate the independent predictors for the red and white microangiopathy group compared to mild microangiopathy (Table 3). Age [per 1 year increase; odds ratio (OR) 1.13; 95% confidence interval (CI) 1.08–1.18; *p*<0.001], and diabetes (OR 2.28; 95% CI 1.02–5.13; *p* = 0.045) were independently associated with white microangiopathy. Other factors, including sex, hypertension, hyperlipidemia, prior history of ischemic stroke or transient ischemic attack, current smoking habit, and alcohol consumption, did not significantly add value to white microangiopathy. In other hand, age (OR 1.05; 95% CI 1.01–1.08; *p* = 0.010) was only independent predictor for red microangiopathy.

**Table 3:** Multiple logistic regression analysis: Independent predictors of red and white microangiopathy.

	Red microangiopathy				White microangiopathy			
	Crude OR(95% CI)	*p*	Multivariate OR (95% CI)	*p*	Crude OR(95% CI)	*p*	Multivariate OR (95% CI)	*p*
Age, per year	1.04 (1.01–1.08)	0.013	1.05 (1.01–1.08)	0.010	1.12 (0.3–1.44)	<0.001	1.13 (1.08–1.18)	<0.001
Male gender	1.46 (0.66–3.22)	0.352			0.82 (0.33–3.83)	0.566		
Hypertension	1.87 (0.88–3.97)	0.105	···	NS	1.91 (0.48–2.02)	0.077	···	NS
Diabetes mellitus	0.59 (0.26–1.37)	0.223			1.72 (0.41–1.66)	0.126	2.28 (1.02–5.13)	0.045
Hyperlipidemia	0.56 (0.23–1.4)	0.218			0.47 (1.07–1.17)	0.098	···	NS
Prior ischemic stroke/TIA	1.24 (0.36–4.26)	0.736			2.64 (0.98–7.14)	0.056	···	NS
Current smoker	0.62 (0.28–1.33)	0.219			0.3 (0.19–1.15)	0.005	···	NS
Heavy alcohol consumption	0.97 (0.42–2.22)	0.938			0.6 (0.25–1.43)	0.251		

TIA indicates transient ischemic attack; NS, non-significant; OR, odds ratio; CI, confidential interval The mild microangiopathy group (n = 112) was used as the reference.

## Discussion

The main finding of this study was that, among patients with acute lacunar stroke, the risk factors were greatly different depending on the concomitant microangiopathic findings. First, white and red microangiopathies commonly coexist in lacunar stroke patients and share common microangiopathic risk factors, such as age, hypertension, and laboratory findings related to viscosity. Second, several risk factors determined the microangiopathic findings on MRI in patients with acute lacunar stroke. Specifically, abnormal glucose metabolism was a risk factor for white microangiopathy, whereas smoking and alcohol habits were risk factors for mild microangiopathic changes; these findings may be important in treatment and prognosis after lacunar stroke.

Although there have been reports about the factors associated with leukoaraiosis and CMB in general and stroke populations, there has been a paucity of data concerning the differential risk factors depending on the MRI subtypes in patients with acute lacunar stroke.

### Lacunar Infarcts that occur in ‘Healthy’ (without other Microangiopathic Findings) Subjects

In the present study, leukoaraiosis or CMB were frequently observed in patients with acute lacunar infarction (leukoaraiosis of Fazekas’ score 3–6 in 42.0% and CMB of 1 or more in 29.2%). Leukoaraiosis were less frequent in this study compared to a previous study of Caucasian lacunar stroke patients, in which the frequency of the Fazekas’ score over 3 was 55.3% [11]. On the contrary, the rate of CMB observed was higher in this study than in a previous meta-analysis, which reported the rate to be 22.9% [12]. This difference may be due to the characteristics of our study population; CMB are prevalent in patients with lacunar stroke, and the prevalence of ICH is reportedly higher in Asians than in Western countries [13].

About half of our patients with lacunar infarction did not have white or red microangiopathic findings. These patients were characterized by a relatively young age, male predominance, and poor control of risk factors, including smoking and alcohol habits. On the contrary, they less frequently had so-called ‘microangiopathic risk factors’ of chronic hypertension and age. This result suggests the importance of lifestyle modification of alcohol and smoking habits to prevent acute lacunar infarction in these patients.

### Common Risk Factors for Both White and Red Microangiopathy

Our data showed that leukoaraiosis and CMB often coexist and that several risk factors (aging, hypertension) and laboratory findings (elevated ESR, fibrinogen) were commonly associated with the severity of both leukoaraiosis and CMB. Moreover, there was a significant correlation between the risk factors and laboratory findings. Therefore, these findings suggest that there are common microangiopathic mechanisms to white and red microangiopathies. Aging and hypertension are generally accepted as microangiopathic risk factors [14]. Fibrinogen was reported to be related with leukoaraiosis and lacunes [15], [16]. ESR was also associated with lacunar infarction [17]. Both fibrinogen and ESR are major determinants of blood viscosity [18]. Our results suggest that blood viscosity is commonly related with microangiopathy, despite the fact that an association between blood viscosity and CMB was not well recognized. Increased blood viscosity may be related with development of microangiopathy by affecting blood flow in resistance vessels and inducing microcirculatory sludging and stagnation in small vessels [19].

We also investigated markers of endothelial dysfunction, because endothelial dysfunction is reportedly associated with lacunar stroke. However, the urine albumin-creatinine ratio was not different according to the severity of microangiopathy in patients with acute lacunar stroke. A recent review showed that current data do not confirm that endothelial dysfunction is specific to small vessel stroke [20].

### Differential Risk Factors between White vs. Red Microangiopathy

Lacunar stroke is a risk factor for ICH as well as the recurrence of lacunar stroke [21]. Although our data showed that the degree of leukoaraiosis and the number of cerebral microbleeds were moderately correlated, about 40% (90 of 230 patients) showed either white or red-predominant subtypes. Leukoaraiosis was associated with recurrence of lacunes [2], whereas CMB were associated with ICH with antithrombotic drug use [4]. These MRI markers may be helpful to predict the long term prognosis and guide the treatment plan in patients with acute lacunar infarctions. Therefore, it is important to know the factors associated with leukoaraiosis-predominant *vs.* CMB-predominant microangiopathy in patients with acute lacunar stroke.

In the present study, diabetes was an independent risk factor for white microangiopathy. It was supported by the findings that serum levels of glucose and hemoglobin A1c were elevated in patients with white microangiopathy. This is in line with a previous longitudinal study that revealed that risk factors for progression of leukoaraiosis are diabetes, blood glucose, and previous stroke [2]. Therefore, it is conceivable that strict control of diabetes is important to prevent progression of white microangiopathy. Further prospective study is needed to verify the effect of glycemic control on prevention of white microangiopathy and subsequent cognitive impairment. It has been previously reported that risk factors for cerebral atherosclerosis, such as hypercholesterolemia and diabetes, and myocardial infarction were more prevalent in patients with lacunar infarcts without leukoaraiosis than in those with leukoaraiosis [11]. However, the weakness of this study is that intracranial vessels were not evaluated, and it is possible that lacunar infarcts could be caused by branch atherosclerotic disease that may mimic SAO [22], [23], [24].

Our results showed that red microangiopathy had similar risk factors to those of the ICH group. Moreover, MRI findings of leukoaraiosis and CMB were similar between the ICH and red microangiopathy subtype. Risk factors for ICH, such as chronic hypertension, old age, and low serum cholesterol, have also been reported to be associated with CMB [25], [26], [27], [28]. Our data failed to find independent risk factors for red microangiopathy except age. That may relate with the dynamic process of CMB after acute stroke [29], [30]. Interestingly, a recent long-term follow-up MRI study reported that most CMB showed dynamic temporal change and that the presence of diabetes was a negatively associated factor for presence of new CMB [30]. Further large cohort studies with serial MRI are needed, and the biological effects of hyperglycemia on brain vascular system need to be clarified.

### Limitations and Conclusions

This study has several limitations. First, although all patients underwent comprehensive workups, including vascular, laboratory, and cardiologic studies, the reader should interpret this study's results with caution given its cross-sectional nature and the limited sample size. Second, although intracranial vessels, as revealed by MRA, might look normal, we cannot rule out the possibility of mild atherosclerotic plaque that did not induce luminal narrowing. One study recently reported that any degree of stenosis, even no demonstrable stenosis, could cause a small, deep infarct extending to the parent artery's basal surface [24]. Thus, the results of this study should be interpreted with caution because of the data from a single center where the prevalence of intracranial atherosclerosis is high. Third, in this study we evaluated various phenotypes of microangiopathy using a high-tesla MRI. However, aside from these visible phenotypes of lacunar stroke, leukoaraiosis, and CMB, there is an emerging concept of MRI ‘non-visible’ expression of SAO [31]. Further studies are needed using quantitative MRI and diffusion-tensor imaging to reveal tissue changes in areas that appear normal on conventional MRI. Lastly, because our definition of subtypes of microangiopathy might be arbituary, we additionally performed analyses with different definitions and the tests showed similar results (data not shown).

In conclusion, patients with acute lacunar infarction had different risk factors according to MRI findings of microangiopathy. A long-term follow-up with serial MRI is needed to evaluate the effects of control of risk factors on differential subtypes of microangiopathy.

## Supporting Information

Table S1 The severity of microangiopathies, risk factors, and laboratory findings.
(DOC)

Table S2 Age and laboratory findings.
(DOC)

Table S3 Hypertension and laboratory findings.
(DOC)
