# Supplementary material for: Differential Risk Factors for Lacunar Stroke Depending on the MRI (White and Red) Subtypes of Microangiopathy
Source: PLoS One. 2012 Sep 14;7(9):e44865. doi: 10.1371/journal.pone.0044865 (PMC3443091; doi:10.1371/journal.pone.0044865)
Supplement: Table S1 — The severity of microangiopathies, risk factors, and laboratory findings. (DOC) [file pone.0044865.s001.doc]

Table S1-3: Correlation analyses of laboratory findings with the severity of microangiopathies and microangiopathic risk factors

Table S1. The severity of microangiopathies, risk factors, and laboratory findings

|  | Fazekas' scale | | CMB | |
| --- | --- | --- | --- | --- |
|  | Correlation coefficient | *p*-value | Correlation coefficient | *p*-value |
| Age | 0.534 | <0.001 | 0.222 | 0.001 |
| ESR, mm/hr | 0.196 | 0.003 | 0.197 | 0.003 |
| Fibrinogen, mg/dL | 0.171 | 0.013 | 0.185 | 0.007 |
| D-dimer, µg/mL | 0.408 | <0.001 | 0.184 | 0.005 |
| Lipoprotein (a), mg/dL | 0.196 | 0.004 | 0.209 | 0.002 |

Spearman’s correlation analyses
