# Supplementary material for: Differential Risk Factors for Lacunar Stroke Depending on the MRI (White and Red) Subtypes of Microangiopathy
Source: PLoS One. 2012 Sep 14;7(9):e44865. doi: 10.1371/journal.pone.0044865 (PMC3443091; doi:10.1371/journal.pone.0044865)
Supplement: Table S2 — Age and laboratory findings. (DOC) [file pone.0044865.s002.doc]

Table S1-3: Correlation analyses of laboratory findings with the severity of microangiopathies and microangiopathic risk factors

Table S2. Age and laboratory findings

|  | Correlation coefficient | *p*-value |
| --- | --- | --- |
| ESR, mm/hr | 0.301 | <0.001 |
| Fibrinogen, mg/dL | 0.201 | 0.003 |
| D-dimer, µg/mL | 0.449 | <0.001 |
| Lipoprotein (a), mg/dL | 0.127 | 0.067 |

Spearman’s correlation analyses
