# Supplementary material for: Differential Risk Factors for Lacunar Stroke Depending on the MRI (White and Red) Subtypes of Microangiopathy
Source: PLoS One. 2012 Sep 14;7(9):e44865. doi: 10.1371/journal.pone.0044865 (PMC3443091; doi:10.1371/journal.pone.0044865)
Supplement: Table S3 — Hypertension and laboratory findings. (DOC) [file pone.0044865.s003.doc]

Table S1-3: Correlation analyses of laboratory findings with the severity of microangiopathies and microangiopathic risk factors

Table S3. Hypertension and laboratory findings

|  | Absence | Presence | *p*-value |
| --- | --- | --- | --- |
| ESR, mm/hr | 15 (7-25) | 18 (9-30) | 0.029 |
| Fibrinogen, mg/dL | 309 (261-356) | 317 (272.5-391) | 0.099 |
| D-dimer, µg/mL | 0.31 (0.22-0.45) | 0.36 (0.26-0.6) | 0.008 |
| Lipoprotein (a), mg/dL | 18.8 (9.2-34.5) | 26.3 (14.2-48.3) | 0.027 |

Mann-Whitney U test

Numbers are median (interquartile range).
